# Reporting checklists are used as measurement tools for assessing quality, even though they have not been validated for such use

**DOI:** 10.1186/s13063-019-3858-6

**Published:** 2019-12-04

**Authors:** Livia Puljak

**Affiliations:** Center for Evidence-Based Medicine and Health Care, Catholic University of Croatia, Ilica 242, 10000 Zagreb, Croatia

Dear Editor,

Kumar Ochani et al. [1] have provided feedback about our manuscript published in *Trials*, in which the reporting quality of randomized controlled trial abstracts was analyzed [2] using the CONSORT for Abstracts (CONSORT-A) checklist [3]. Kumar Ochani et al. [1] have rightfully pointed out some limitations of that work, namely the fact that we have used the “total adherence score” for presenting adherence to the reporting checklist, which gives equal weight to each item, and the lack of measures of inter-rater reliability.

Admittedly, in this study and in many similar studies employing this or another reporting checklist with similar aim, a reporting guideline was used as a measurement tool for assessing the reporting quality. Reporting checklists have been developed as guidelines for adequate reporting of a certain type of study, and they were not developed as validated measurement tools, or validated for this purpose subsequently. Therefore, we have a situation in which many authors use reporting checklists for assessing the reporting quality of research-related reports with something that is not a measurement tool.

For practical reasons, many authors have resorted to both presenting results for adherence to individual reporting checklist domains as well as presenting an adherence score – for example, using scores of 0 points for “no adherence”, 0.5 points for “partial/unclear adherence” and 1 point for “adherence”. Calculating an adherence score like this indeed results in a potentially unfair situation in which all items are weighted equally in a total adherence score. However, by providing an adherence score for each individual item in addition to a total adherence score, readers can get a transparent picture of compliance with each item, and they can see which items are more neglected than others in terms of reporting quality.

Since many authors are using reporting checklists as measurement tools for assessing reporting quality of studies, research efforts that would formally explore and validate usage of those checklists as measurement tools would be welcome. Such studies would result in formal guides with steps that should be taken for an assessment to be considered adequate. For example, such guidance could stipulate that the assessment of reporting quality always has to be made by two authors independently, that inter-rater agreement between the authors is a mandatory part of such an assessment (with exact methods specified), and that subgroup analyses need to be presented for each analyzed source to enable comparisons (for example each analyzed journal).

Without such guidance, the way authors use reporting checklists as measurement tools will remain non-validated and arbitrary, and will continue to depend on personal preferences.

## Data Availability

Not applicable.
